# Olfactory discrimination in disorders of consciousness: A new sniff protocol

**DOI:** 10.1002/brb3.1273

**Published:** 2019-06-28

**Authors:** Davide Sattin, Maria Grazia Bruzzone, Stefania Ferraro, Anna Nigri, Matilde Leonardi, Davide Guido, Coma Research Center, Fondazione IRCCS Istituto Neurologico “Carlo Besta”, Milan, Italy

**Affiliations:** ^1^ Neurology, Public Health and Disability Unit, Coma Research Center Fondazione IRCCS Istituto Neurologico Carlo Besta Milan Italy; ^2^ Neuroradiology Department Fondazione IRCCS Istituto Neurologico “Carlo Besta” Milan Italy

**Keywords:** Bayesian approach, disorders of consciousness, neuroimaging, olfactory test, vegetative state

## Abstract

**Background:**

The identification of salient stimuli useful for rehabilitation purposes is important in patients with disorders of consciousness (DOC): among these, olfactory stimuli might play an important role due to the functional coupling between olfactory and emotional processing. However, a high percentage of post brain injury patients present anosmia.

**Aims of the Study:**

The aim of this pilot research is to present an innovative approach to test olfactory functions at the bedside using four selected odors in patients with DOC.

**Methods:**

Sixteen patients with DOC were tested with two assessment techniques the new olfactory discrimination protocol (ODP) and a functional magnetic resonance imaging paradigm to evaluate olfactory neural process. The Frequentist and Bayesian methods were used to analyze reliability properties of the new tool.

**Results:**

Analysis showed a good agreement between assessment techniques and a substantial test‐retest reliability of the ODP. Cohen's Ks were equal to 0.814 (95% CI = 0.471, 1) and 0.607 (0.118; 1) respectively, using the Frequentist approach, while they were 0.762 (95% HPD = 0.470; 0.966) and 0.650 (0.320; 0.913) with the Bayesian approach in the 11 patients analyzed.

**Conclusions:**

Despite the limits of this preliminary research, the ODP can be useful for clinicians for the preliminary assessment of the olfactory discrimination in patients with DOC.

## 1 INTRODUCTION

The sense of olfaction is functionally linked to emotional processes and it has particular anatomical features respect to other senses. Indeed, it is not subordinated to a full direct thalamic relay, and odor signals intersect the olfactory bulb toward the piriform cortex, the lateral‐medial orbitofrontal cortex (lOFC), and other subcortical structures such as the amygdala (Nigri et al., 2013). However, the probability of a patient having hypo/anosmia following a brain injury is quite high (Proskynitopoulos, Stippler, & Kasper, 2016). Patients with Disorders of consciousness (DOC) do not show or show only limited signs of conscious behaviors (The Multi‐Society Task Force on PVS, 1994), and their assessment and rehabilitation are a challenge for clinicians. Professionals evaluate behavioral responses after multi‐sensorial inputs and to identify salient items that may be helpful for planning interventions using, for example, Operant Conditioning Paradigms (Lancioni et al., 2009). The aim of this brief pilot research study is to present an innovative test for olfactory function in patients with DOC at the bedside, assessing the test‐retest reliability of this tool and the agreement with a dichotomous functional magnetic resonance imaging (fMRI) indicator (suggesting the presence of preserved olfactory neural processing).

## 2 PATIENTS AND METHODS

Sixteen patients diagnosed with DOC were recruited during the “Coma Research Center” program. Inclusion criteria were: (a) diagnosis of DOC defined with the Coma recovery scale‐revised (CRS‐R) (Giacino, Kalmar, & Whyte, 2004; Sattin et al., 2015), (b) no signs of degenerative neurological diseases previous to the acute event, and (c) loss of consciousness for more than 6 hr with a Glasgow Coma Scale scores ranged between 3 and 8 after the acute event. Data on olfactory problems before the acute event were collected in two ways: asking caregivers/familiars if they were aware of patient olfactory problems and checking the electronic clinical record. The presence of olfactory problems as well as nasal airway obstructions after severe facial trauma were used as pre‐defined exclusion criteria.

This research was approved by the Fondazione IRCCS Istituto Neurologico C. Besta ethics committee and informed written consent was obtained from patients' representatives.

### 2.1 fMRI data acquisition and protocol

Functional magnetic resonance imaging methods and data of these patients are reported in a previous paper investing a wider sample (Nigri et al., 2016). Odors (1‐octenol‐3‐ol and n‐butanol, i.e., odor quality of mushroom and white‐board marker, respectively) were presented using an MRI‐compatible computer‐controlled olfactometer. The imaging data were acquired using an Achieva 3‐T MR scanner (Philips Healthcare, Best, NL) equipped with a 32‐channel head coil. Briefly, analyses were performed using Statistical Parametric Mapping (SPM8) and standard fMRI data pre‐processing. The pre‐processed functional data for each participant were entered in single‐subject whole‐brain analyses. Odor‐related neural activations were determined by the contrast of the regressors “odor > baseline”. Two functional regions of interest were identified: the piriform cortex extending into the amygdala cortex (PC/AMY) and the lateral orbito‐frontal cortex (lOFC) (voxel‐level threshold = *p* < 0.005 uncorrected; minimum cluster size = 20 voxels), two areas important for the primary and the higher order olfactory processing respectively (Nigri et al., 2016). Each patient was assigned to one of the following categories according to the presence or absence of activations in response to the odor stimuli in the previously defined ROIs (PC/AMY and lOFC): no activation, activation in PC/AMY, and activation in PC/AMY and lOFC). As reported in the previous study, we were interested in evoking robust neural responses; therefore we preferred not to reduce the statistical power of our analyses modelling the two odorants as different regressors (reducing the degrees of freedom of our model). It is important to stress that we used two different stimuli during the fMRI session in order to avoid the known problem of habituation during the olfactory processing. Importantly, in the previous study, 28 healthy volunteers participated in the neuroimaging session of the experiment in order to validate the fMRI protocol.

### 2.2 The ODP for patients with DOC

Four kinds of odors were tested twice in a week during the CRS‐r assessment by the same expert rater (defined as a rater with several years of experience and who has assessed more than 30 patients with the CRS‐r) (Løvstad et al., 2010). The Olfactory discrimination protocol (ODP) was composed of four odors selected and dosed according to the literature on clinical sniff tests (Frank, Dulay, & Gesteland, 2003; Kobal et al., 2001; Murata et al., 2007) and each nostril was tested. Odorants were presented randomly in commercially available test‐tubes with a length of approximately 12 cm, and with an inner diameter of 1.4 cm: (a) one pen contained 99% of Phenyl ethyl alcohol solution (a rose‐like odor), that is hereafter called “Positive stimulus”; (b) another one contained S‐methyl thiobutanoate, 98% purity, in mineral oil (1.0% vol/vol), (described as having a fecal, putrid, decaying, rancid odor) called “Negative stimulus 1”; (c) one pen was filled with 13.5% isovaleric acid diluted in propylene glycol (smell of sweat, stuffy socks) called “Negative stimulus 2”; finally, (d) one pen contained Ammonia 366 ppm solution evoking trigeminal‐mediated irritation of the nose, called the “Trigeminal stimulus”. This last stimulus was chosen as a control stimulus in order to evaluate the behavioral responses to an irritative stimulation of nasal mucosa. In detail, the trigeminal stimulus was used to test if a patient was able (or not) to move his/her own head in order to avoid the irritating stimulation of the nasal mucosa. The head movement of avoidance is expected by the rater as a normal/innate behavioral response to irritant stimuli. The absence of head movement of avoidance during the administration of the “Trigeminal stimulation” was considered as evidence of the probable presence of (e.g.,) motor or nociceptive impairment. This is important information that allows the reduction in false negatives, considering that negative stimuli present a weaker property to evoke a head movement (the object of the behavioral observation) other than the trigeminal stimulation.

Importantly, ammonia was chosen in relation to its higher commerce availability more than other substances (e.g., CO_2_) and also for the confidence of the clinicians with its use (ammonia was used for several years during the clinical assessment with the Coma Near Coma scale (Rosazza et al., ), an assessment tool that was commonly used for the evaluation of persons with DOC before the CRS‐R development). Ammonia threshold was determined considering previous studies (Geisler & Murphy, 2000; Petrova, Diamond, Schuster, & Dalton, 2008).

Before the odor presentation, the clinician put a mirror under the nose of each patient to test the presence of airflow from the nose. In patients with tracheostomy, this test was performed twice, closing the tracheostomy tube for 10 s (patients' oxygen saturation was monitored during the procedure). For odor presentation the cap was removed by the experimenter for approximately 3s and the pen's tip was placed 2–3 cm in front of left (and then the right) nostrils. A 15 s interstimulus interval within the same odor presentations was imposed, whereas a 2‐minute interval was used between different odors. Clinician meanwhile, used a portable fan to vanish residual odors in the air between pens presentation.

### 2.3 Statistical analysis

Two dichotomous (present/absent) indicators were developed, one for the ODP and one for the previously published fMRI data (Nigri et al., 2016). For the ODP: behavioral responses (eyes closure, grimace, avoiding head movement, or vocalization) in correspondence to odor presentation (<10 s) were classified according to the following scheme: 1 = Presence of behavioral response both after trigeminal stimulation and at least one negative stimulus but not after positive stimulus presentation, 0 = indiscriminated behavioral responses or presence of response also for positive stimulus. The best performance ODP indicator was determined by the best performance value between test and re‐test. For the fMRI, functional activity was classified according to the following scheme: we assigned value = 1 when we detected activation of the PC/AMY and the OFC, while value = 0 when we detected activations only in PC/AMY or no activation.

Descriptive statistics on gender (frequency and percentage) and age (median value and Interquartile range) were used. In order to evaluate the agreement between assessment techniques the Cohen's Kappa (K) was computed. In addition, the K was also used to evaluate the test re‐test reliability of the ODP (within a week in relation to the hospitalization time of the Project funding this research). The K was estimated by using both Frequentist (traditional) and Bayesian approach, as suggested Lee and Wagenmakers (2013), that can provide more accurate estimates for small samples (McNeish, 2016). ODP diagram (S1) and the Bayesian methodology (S2) are available in the Data S1 and Data S2.

## 3 RESULTS

A total of 16 patients were involved in the study. Four of them were excluded from the final analysis due to head movements during fMRI data acquisition, and one patient was not considered during the acquisition of data because he showed inconsistent airflow from the nose (and he also presented tracheostomy).

**Figure 1:**
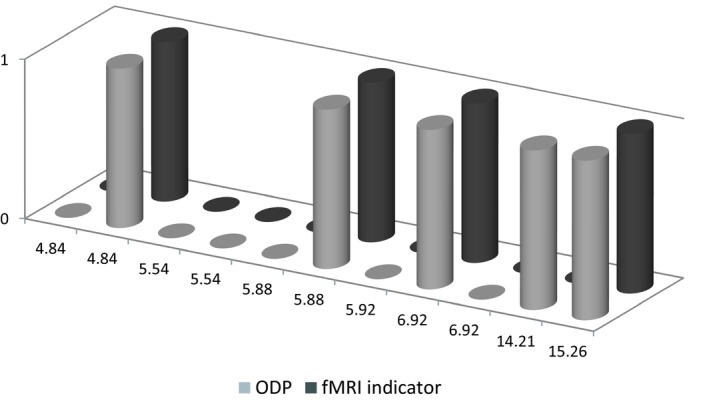
Graphical representation of the olfactory discrimination protocol (ODP) indicators and the functional magnetic resonance imaging (fMRI) indicators in relation to Coma recovery scale‐revised modified scores obtained during clinical assessment

Among the 11 patients analyzed six (54.5%) were female, and the median age was 57 years old (IQR = 13.5). Clinical features, ODP and fMRI results were reported in Table 1 and Figure 1. Eleven patients (91.6%) obtained the same scores in ODP and fMRI indicators.

**Table 1:** Patients clinical characteristics, ODP indicator, and fMRI olfactory neural activation in DOC patients

Diagnosis/ID	Tracheostomy tube	CRS‐r/CRS‐r MS scores	Duration of disease	ODP indicator	Odor‐related neural activation fMRI indicator	Aetiology	PC/AMY		lOFC	
	(yes/no)		(months)	(0,1)	(0, 1)		R	L	R	L
VS/UWS1	yes	8/5.92	10	0	0	ABI	A	A	A	A
VS/UWS2	yes	6/5.54	3	0	0	ABI	—	—	—	—
VS/UWS3	no	7/5.88	58	0	0	HBI	—	—	—	—
VS/UWS4	yes	8/6.92	5	1	1	HBI	✓	—	✓	—
VS/UWS5	yes	6/4.84	9	0	0	ABI	A	A	A	A
VS/UWS6	no	6/5.54	8	0	0	ABI	—	—	—	—
VS/UWS7	yes	7/5.88	20	1	1	HBI + IBI	—	✓	✓	✓
VS/UWS8	no	6/4.84	16	1	1	ABI	—	✓	✓	✓
VS/UWS9	yes	8/6.92	25	0	0	TBI + ABI	—	✓	—	—
MCS1	yes	9/15.26	49	1	1	HBI	NC	✓	A	✓
MCS2	yes	8/14.21	146	1	0	ABI	A	A	A	A

Note

Degree of preservation of odor‐related neural activation according to the presence or the absence of activation in PC/AMY and lOFC in single‐subject analyses (fMRI indicator = 0: no activation or partial activation in PC/AMY; fMRI indicator = 1: activation in PC/AMY and lOFC); ODP indicator derived from ODP assessment (ODP = 0: indiscriminate behavioral responses or presence of response also for positive stimulus; ODP = 1: presence of behavioral response both after trigeminal stimulation and at least one negative stimulus but not after positive stimulus presentation).

✓: significant activation within the region; A: atrophic region; NC: not classified, region not identified; TBI: traumatic brain injury; HBI: hemorrhagic brain injury; ABI: anoxic brain injury; IBI: ischemic brain injury; PC/AMY: piriform cortex/amygdala; lOFC: lateral‐medial orbitofrontal cortex; CRS‐r: Coma recovery scale revised; CRS‐r MS: Coma recovery scale revised modified score; ODP: Olfactory discrimination protocol; R: right hemisphere; L: left hemisphere; fMRI: functional magnetic resonance imaging.

In table 2 K values were reported. Concerning the frequentist approach, the K returned a good value equal to 0.814 (95% CI = 0.471; 1) for agreement and a substantial 0.607 (0.118; 1) for test re‐test reliability. The results were also computed by bootstrap resamples to compare the values obtained by the Frequentist approach (traditional way). Following the Bayesian approach, the model 3, with optimistic priori distribution, returned the best fit (agreement: deviance information criterion (DIC) = 11.1; test re‐test reliability DIC = 12.4), whose Ks were 0.762 (95% highest posterior density (95% HPD) interval = 0.470; 0.966) and 0.650 (0.320; 0.913) respectively (see S2). Other results are provided in the Data S1 and Data S2.

**Table 2:** Agreement analysis between the ODP and the fMRI olfactory protocol and test re‐test reliability of the ODP in patients with disorders of consciousness

		Contingency tables [absolute frequencies and (relative frequencies)]			Frequentists methods		Bayesian methods	
					Cohen's K	*95% CI*	Cohen's K	*95% HPD*
Agreement analysis (*ODP* *vs* *fMRI indicators*)								
			ODP indicator					
			*Absent*	*Present*				
	fMRI indicator	*Absent*	6 (0.545)	1 (0.091)	0.814	*0.471; 1*	0.762	*0.470; 0.966*
		*Present*	0 (0)	4 (0.363)				
Test re‐test reliability (ODP *test1* vs. *test2*)								
			ODP (*test 2*)					
			*Absent*	*Present*				
	ODP (*test 1*)	*Absent*	6 (0.545)	1 (0.091)	0.607	*0.118; 1*	0.650	*0.320; 0.913*
		*Present*	1 (0.091)	3 (0.273)				

Note

K values reference: values <0 as indicating no agreement and 0–0.20 as slight, 0.20–0.40 as fair, 0.40–0.60 as moderate, 0.60–0.80 as substantial, and 0.80–1 as almost perfect agreement.

ODP: Olfactory discrimination protocol; CI: Confidence interval; HPD: Highest posterior density interval; fMRI: functional magnetic resonance imaging.

## 4 DISCUSSION

To our knowledge, this pilot research is the first study to test the olfactory discrimination in patients with DOC at the bedside. ODP for patients with DOC showed substantial test‐retest reliability and an almost perfect agreement with a dichotomous fMRI indicator (suggesting the presence of preserved olfactory neural processing). The advantages of ODP are the time of approximately 5 min needed for its application and the possibility to perform a bedside evaluation.

It is widely accepted that the assessment of sensory processes could be very useful in several neuropathologies and the analysis of the olfactory sense can be useful both for preclinical diagnosis (e.g., in Parkinson disease) (Maremmani et al., 2018) and for rehabilitation purposes (e.g., for the selection of odors that potentially can be used to exert positive influences in classical or operating conditioning rehabilitation paradigms).

As reported in the introduction, the sense of olfaction is not dependent from a direct thalamic relay and the olfactory receptors are implicated in saliency processing and memory (involving amygdala, hippocampus, etc.). On the basis of these characteristics, the olfactory sense could be the most direct way that clinicians and caregivers could use to stimulate memory and emotions in patients with DOC. Unfortunately, we have no information on the elaboration of olfactory stimuli in these patients nowadays, because it is not usually evaluated during common clinical practice. The ODP can be a useful tool to fill this gap, although we stressed the idea that the ODP can only detect the basic behavioral reactions to different olfactory stimuli as an indirect evidence of a process linked to the olfactory discrimination. This assumption is fundamental, and ODP cannot provide information suggesting advanced cognitive elaboration of the olfactory stimuli at the moment.

The difference between ODP and other clinical tool used for assessing olfactory sense in patients with DOC, as the Coma Near Coma (CNC) (Rappaport et al., 2005) or the DOC‐25 (Pape, Mallinson, & Guernon, 2014), is related to the different aims of these tests: the CNC tests the behavioral responses to ammonia in a single item and it does not analyze discrimination between different odors, whereas in the DOC‐25 olfactory evaluation is more related to taste sense than to a discrimination analysis.

We acknowledge the limitations of our study. First, the sample size was relatively small and this could cause a selection bias, although this pilot study was designed for DOC patients as population of interest (Kottner et al., 2011). Second, it is well known that odors and trigeminal responses could be affected by a number of non‐sensory factors like personality, beliefs about hazards from chemicals, etc. (Petrova et al., 2008). However, in this research the highest concentrations published for each odor were used in order to avoid a sub‐threshold stimulation. Third, the choice of only four odors could underestimate the real olfactory functions (high risk to specific anosmia) than a composite analysis, although ODP represents a good easy tool to pretest noncommunicative patients untestable with classical tools. Finally, the fMRI study of these patients is quite difficult due to their head movement. However, the percentage of excluded patients in this pilot research was in line with previous studies, as reported in a relatively recent work which showed that 62 patients out of 119 presented large head movements during the resting state fMRI (Rosazza et al., ). Moreover, the four patients excluded showed movement related mainly to the noise of the MRI rather than to olfactory stimuli.

An interesting issue for possible future research could be the correlation between the ODP results and the quantitative analysis of the nasal airflow (e.g., the airflow resistance or the volume of air exhaled through the nostrils). After the development of a normative values table, this analysis could have an important impact in clinical practice because it could orient clinicians to analyze problems related to the respiratory system that, otherwise, remain without a diagnosis in these patients. However, future studies are needed in order to test ODP in a wider sample than those involved in this pilot study. Moreover, it will be useful to test other odors in order to determine what is the best subset of odors to determine a good sensibility and specificity in relation to fMRI results.

In conclusion, although future studies are needed, the presented protocol could be a useful, fast, and easy tool to test olfactory sense in patients with DOC.

## CONFLICTS OF INTEREST

The authors declare no financial or other conflicts of interest.
