# Supplementary material for: Olfactory discrimination in disorders of consciousness: A new sniff protocol
Source: Brain Behav. 2019 Jun 28;9(8):e01273. doi: 10.1002/brb3.1273 (PMC6710199; doi:10.1002/brb3.1273)
Supplement: Supplementary file 1 [file BRB3-9-e01273-s001.doc]

**OLFACTORY DISCRIMINATION PROTOCOL**

for patients with Disorders of consciousness

**First Name______________________ Last name__________________________**

**Tracheostomy tube O**  yes **O** no

**Nasal airflow O** yes  **O** no

CRS-r score_______

| **TEST n°1** | **1** | | **2** | | **3** | | **4** | |
| --- | --- | --- | --- | --- | --- | --- | --- | --- |
|  | R | L | R | L | R | L | R | L |
| **Positive stimulus** |  | |  | |  | |  | |
| PEA |  |  |  |  |  |  |  |  |
| **Negative stimulus 1** |  | |  | |  | |  | |
| MTB 1% |  |  |  |  |  |  |  |  |
| **Negative stimulus 2** |  | |  | |  | |  | |
| IA |  |  |  |  |  |  |  |  |
| **Trigeminal stimulus** |  | |  | |  | |  | |
| AMM |  |  |  |  |  |  |  |  |

Note:____________________________________________________________________________

CRS-r score_______

| **TEST n°2** | **1** | | **2** | | **3** | | **4** | |
| --- | --- | --- | --- | --- | --- | --- | --- | --- |
|  | R | L | R | L | R | L | R | L |
| **Positive stimulus** |  | |  | |  | |  | |
| PEA |  |  |  |  |  |  |  |  |
| **Negative stimulus 1** |  | |  | |  | |  | |
| MTB 1% |  |  |  |  |  |  |  |  |
| **Negative stimulus 2** |  | |  | |  | |  | |
| IA |  |  |  |  |  |  |  |  |
| **Trigeminal stimulus** |  | |  | |  | |  | |
| AMM |  |  |  |  |  |  |  |  |

Note:____________________________________________________________________________

| **Best Performance score** | **1** | | **2** | | **3** | | **4** | |
| --- | --- | --- | --- | --- | --- | --- | --- | --- |
|  | R | L | R | L | R | L | R | L |
| Positive stimulus (PS) |  |  |  |  |  |  |  |  |
| Negative stimulus (NS1) |  |  |  |  |  |  |  |  |
| Negative stimulus 2 (NS2) |  |  |  |  |  |  |  |  |
| Trigeminal stimulus (TS) |  |  |  |  |  |  |  |  |
|  |  |  |  |  |  |  |  |  |
| **Behavioral Response after TS** | **[ ] yes** | | | | **[ ] no** | | | |
| **Discrimination between PS and NS1 and/or NS2** | **[ ] yes** | | | | **[ ] no** | | | |

*Legend:R, L=right and left nostrils; PEA Phenyl ethyl alcohol; MTB S-methyl thiobutanoate; IA isovaleric acid; AMM Ammonia. Response code: A: eyes closure; B: grimace; C:avoiding behavior/head movement; D:vocalization; E: other response).*
