# Supplementary material for: Olfactory discrimination in disorders of consciousness: A new sniff protocol
Source: Brain Behav. 2019 Jun 28;9(8):e01273. doi: 10.1002/brb3.1273 (PMC6710199; doi:10.1002/brb3.1273)
Supplement: Supplementary file 2 [file BRB3-9-e01273-s002.docx]

**Bayesian analysis**

*Methods*

We have followed the Bayesian approach by performing a full Bayesian analysis by Markov chain Monte Carlo (MCMC). The median of the κ posterior distribution (by MCMC) with respective 95% highest posterior density (95%HPD) intervals was computed as point estimation (for both agreement and test re-test reliability). In addition, standard deviations and Monte Carlo errors of posterior distributions were also computed to further validate the estimates. Data were processed in aggregate format as contingency table (2x2 table) and they followed a multinomial probabilistic model (i.e. likelihood function) as reported in the Jags code (y[1:4] ~ dmulti(pi[],n)) at the end of this supplementary material. With this approach κ is considered as a coefficient of chanche-corrected agreement (or test re-test reliability). Hence, we also estimated and monitored two other relevant parameters, such components of κ: the raw agreement (or test re-test reliability) coefficient (ξ) and the coefficient of agreement (or test re-test reliability) that would occur by chance (ψ) [1]. Moreover, since we have worked in a Bayesian framework, we have assessed three different scenarios of prior distributions by recording all the parameter estimates. Consequently, we performed a sensitivity analysis by DIC (deviance information criterion, the lowest was better) for the choice of the prior, by trying a range of plausible distributions. In this way, sceptical, vague and optimistic priors were elicited on the two concordant cell rates: (i) Beta(1,5) (model 1), (ii) Beta(1,1) (model 2) and (iii) Beta(5,1) (model 3) (see bayesian models in Appendix). Finally, a Beta(2,7) distribution prior was elicited on the rate of non-response obtained by fMRI indicator (standard assessment for the agreement) or on the rate of non-response obtained by first test (in the test re-test reliability), following the info provided by de Kruijk et al. [2].

Accounting for MCMC algorithms, the results were based on 250,000 iterations after a burn-in period of 1,000 iterations. The accuracy of the results has been assessed by convergence of MCMC algorithm, that were checked both using graphical inspection of running means, trace plots and using all methods included in R/boa package [3].

Statistical analyses were performed by Jags [4], R [5] and its packages psych [6], R2jag [7], boa [3] and coda [8].

*Results*

Regarding bayesian analysis, the convergence of the MCMC posterior distributions was achieved (see plots of the diagnostics in Figure 1S). The estimation of κ varied across scenario (coded by different prior distributions, see Figure 2S) and the model 3 provided the best results (compared with the cut-offs suggested by Landis et al. [9]) and fit: agreement κ=0.762 (DIC=11.1), test re-test reliability κ =0.650, (DIC=12.4)(see table 1S). However, also the model 2, whose priori distribution reprsented lack of priori knowledge, returned acceptable results in both terms of agreement (κ = 0.629) and test re test reliability (κ = 0.442). In addition, as expected, the ξ coefficients was always bigger than κ being its raw version, and the ψ was estimated close to 0.5 because was hypothesed as the coefficient of agreement that occured by chance. Moreover, the model 3 returned the worst fit and κ values were close to 0, which implies that there were no effective agreement and test re- test reliability and the they were worse than those occured by chance (ψ).

Finally, it is worth to point out that the bayesian estimates was influenced by prior distribution in this case of small sample size study.

**Table 1S. Results of the Bayesian analysis**

| Property | Parameter | Model 1 (with sceptical prior: Beta(1,5)) | | | | Model 2 (with vague prior: Beta(1,1)) | | | | **Model 3 (with optimistic prior: Beta(5,1))** | | | |
| --- | --- | --- | --- | --- | --- | --- | --- | --- | --- | --- | --- | --- | --- |
|  |  | Median | SD | 95%HPD | MC error | Median | SD | 95%HPD | MC error | **Median** | **SD** | **95%HPD** | **MC error** |
| Agreement | κ | 0.036 | 0.191 | -0.336; 0.409 | 0.001 | 0.629 | 0.197 | 0.219; 0.939 | 0.001 | **0.762** | **0.137** | **0.470; 0.966** | **0.001** |
|  | ξ | 0.518 | 0.105 | 0.311; 0.716 | 0.000 | 0.823 | 0.100 | 0.612; 0.975 | 0.000 | **0.887** | **0.068** | **0.744; 0.987** | **0.000** |
|  | ψ | 0.500 | 0.027 | 0.441; 0.559 | 0.000 | 0.508 | 0.036 | 0.466; 0.608 | 0.000 | **0.511** | **0.035** | **0.484; 0.607** | **0.000** |
|  | DIC | 26.0 | | | | 13.6 | | | | **11.1** | | | |
|  |  |  |  |  |  |  |  |  |  |  |  |  |  |
| Test re-test  reliability |  | Median | SD | 95%HPD | MC error | Median | SD | 95%HPD | MC error | **Median** | **SD** | **95%HPD** | **MC error** |
|  | κ | -0.059 | 0.186 | -0.425; 0.305 | 0.001 | 0.442 | 0.220 | 0.008; 0.836 | 0.001 | **0.650** | **0.159** | **0.320; 0.913** | **0.000** |
|  | ξ | 0.461 | 0.104 | 0.259; 0.664 | 0.000 | 0.727 | 0.117 | 0.488; 0.928 | 0.000 | **0.832** | **0.081** | **0.659; 0.959** | **0.000** |
|  | ψ | 0.497 | 0.031 | 0.417; 0.548 | 0.000 | 0.502 | 0.033 | 0.437; 0.581 | 0.000 | **0.506** | **0.030** | **0.471; 0.587** | **0.000** |
|  | DIC | 22.7 | | | | 13.8 | | | | **12.4** | | | |

κ: Cohen’s Kappa (chanche-corrected agreement): ξ: the raw coefficient of agreement; ψ: coefficient of agreement that would occur by chance; SD: Standard Deviation; 95%HPD: 95% Highest Posterior Density interval; MC error: Monte Carlo error gives an estimate of the Monte Carlo standard error of the mean; DIC: Deviance Information Criterion. In **bold** the best bayesian model (i.e. the lowest DIC).

**Figure 1S. Diagnostics of the bayesian analysis**

*Model 1 – agreement Cohen’s Kappa (with sceptical Beta priors on the two concordant cell rates)*


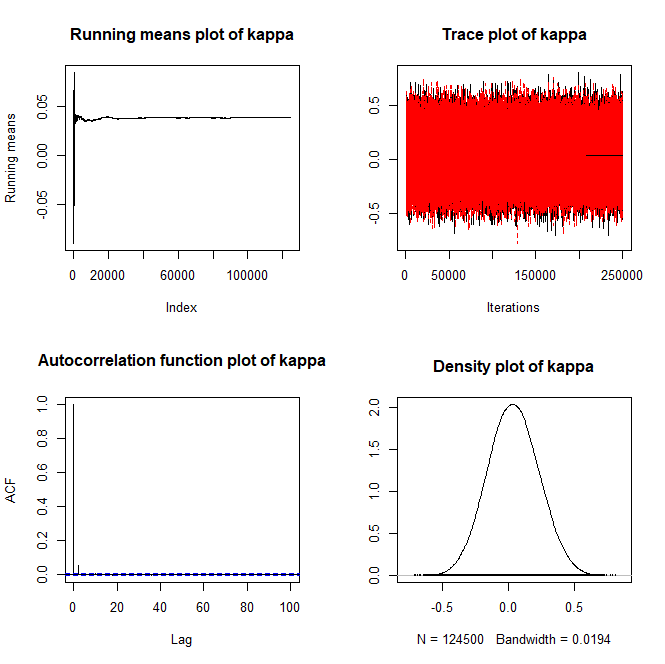


*Model 1 – test re-test reliability Cohen’s Kappa (with sceptical Beta priors on the two concordant cell rates)*


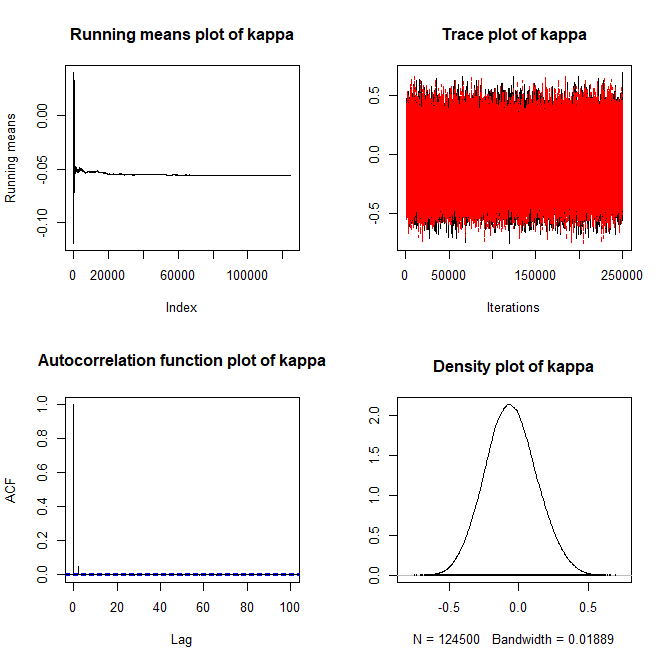


*Model 2 – agreement Cohen’s Kappa (with vague Beta priors on the two concordant cell rates)*


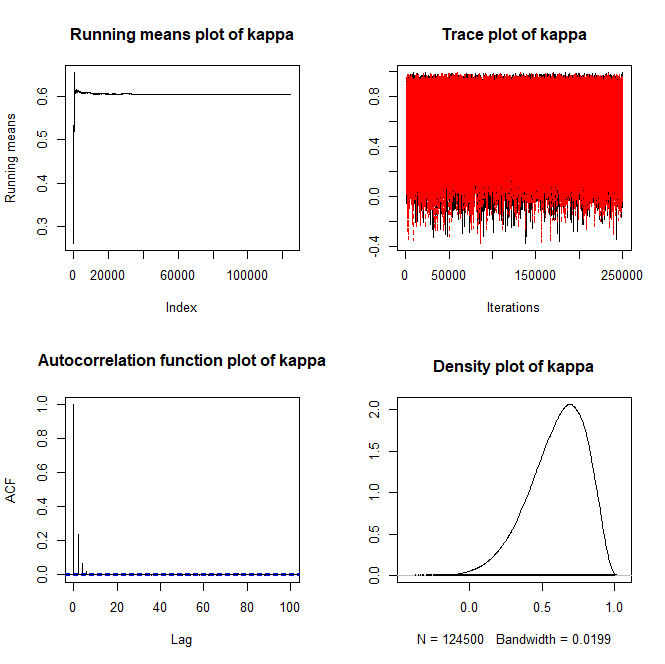


*Model 2 – test re-test reliability Cohen’s Kappa (with vague Beta priors on the two concordant cell rates)*


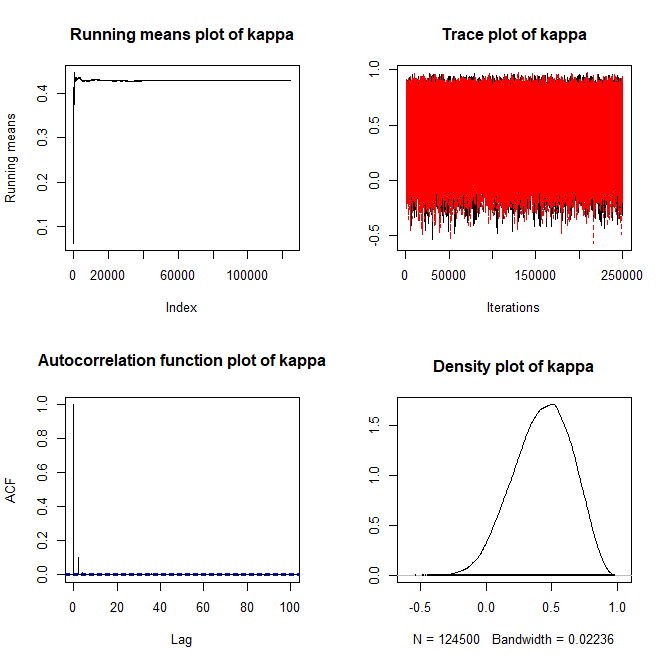


*Model 3 - agreement Cohen’s Kappa (with optimistic Beta priors on the two concordant cell rates)*


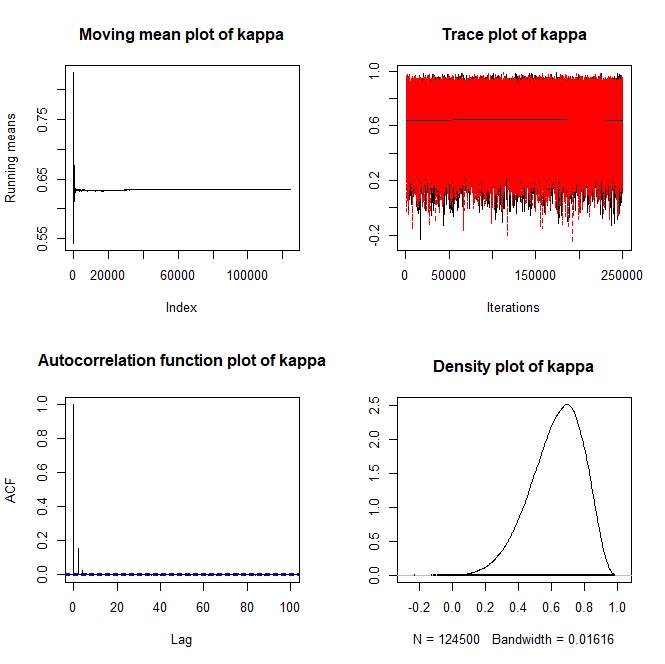


*Model 3 – test re-test reliability Cohen’s Kappa (with optimistic Beta priors on the two concordant cell rates)*


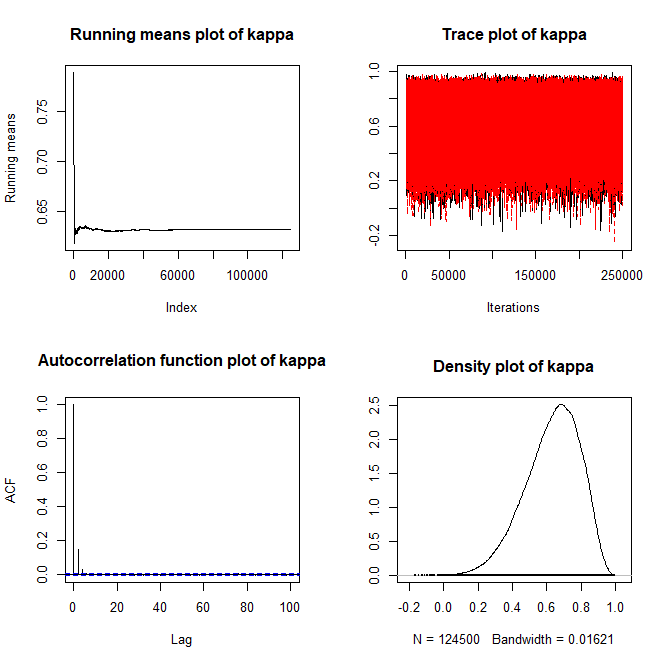


**Figure 2S. Beta priors distributions**
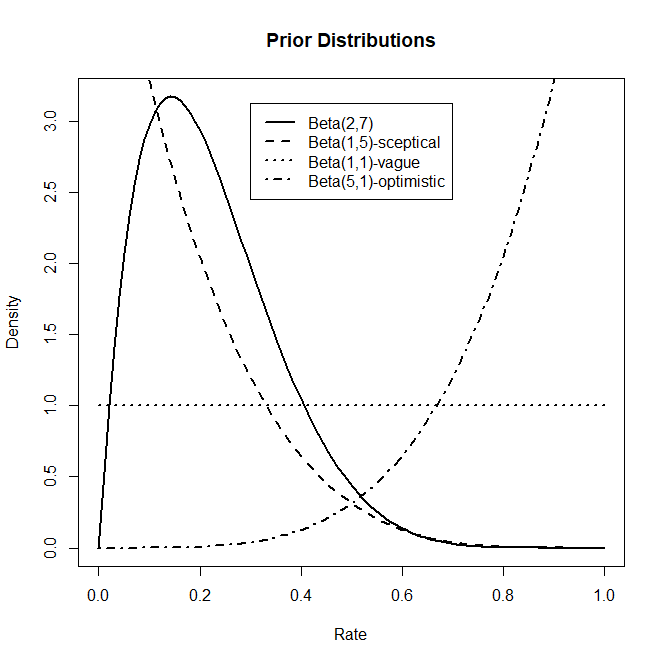


**Appendix - Bayesian models**

*The model 1 (with sceptical Beta priors on the two concordant cell rates)*

# Kappa Coefficient (agreement or test re-test reliability)

model{

# Underlying Rates

# Rate “non-respondents” by fMRI indicator (agreement)

# Rate “non-respondents” by first test by ODP (test re-test reliability)

alpha ~ dbeta(2,7)

# Rate ODP decides "non-respondent" when fMRI indicator decides "non-respondent" (agreement)

# Rate re-test decides "non-respondent" when test decides "non-respondent" (test re-test reliability)

beta ~ dbeta(1,5)

# Rate ODP decides "respondent" when fMRI indicator decides "respondent"

# Rate re-test decides " respondent" when test decides " respondent" (test re-test reliability)

gamma ~ dbeta(1,5)

# Probabilities For Each Count

pi[1] <- alpha*beta

pi[2] <- alpha*(1-beta)

pi[3] <- (1-alpha)*(1-gamma)

pi[4] <- (1-alpha)*gamma

# Count Data

y[1:4] ~ dmulti(pi[],n)

# Derived Measures

# Rate of ODP agrees with the fMRI indicator (agreement)

# Rate of re-test agrees with the test (test re-test reliability)

xi <- alpha*beta+(1-alpha)*gamma

# Rate of Chance Agreement (agreement)

# Rate of Chance Test re-test reliability (test re-test reliability)

psi <- (pi[1]+pi[2])*(pi[1]+pi[3])+(pi[2]+pi[4])*(pi[3]+pi[4])

# Chance-Corrected Agreement (agreement)

# Chance-Corrected Test re-test reliability (test re-test reliability)

kappa <- (xi-psi)/(1-psi)

}

*The model 2 (with vague Beta priors on the two concordant cell rates)*

# Kappa Coefficient (agreement or test re-test reliability)

model{

# Underlying Rates

# Rate “non-respondents” by fMRI indicator (agreement)

# Rate “non-respondents” by first test by OPD (test re-test reliability)

alpha ~ dbeta(2,7)

# Rate ODP decides "non-respondent" when fMRI indicator decides "non-respondent" (agreement)

# Rate re-test decides "non-respondent" when test decides "non-respondent" (test re-test reliability)

beta ~ dbeta(1,1)

# Rate ODP decides "respondent" when fMRI indicator decides "respondent" (agreement)

# Rate re-test decides " respondent" when test decides " respondent" (test re-test reliability)

gamma ~ dbeta(1,1)

# Probabilities For Each Count

pi[1] <- alpha*beta

pi[2] <- alpha*(1-beta)

pi[3] <- (1-alpha)*(1-gamma)

pi[4] <- (1-alpha)*gamma

# Count Data

y[1:4] ~ dmulti(pi[],n)

# Derived Measures

# Rate of ODP agrees with the fMRI indicator (agreement)

# Rate of re-test agrees with the test (test re-test reliability)

xi <- alpha*beta+(1-alpha)*gamma

# Rate of Chance Agreement (agreement)

# Rate of Chance Test re-test reliability (test re-test reliability)

psi <- (pi[1]+pi[2])*(pi[1]+pi[3])+(pi[2]+pi[4])*(pi[3]+pi[4])

# Chance-Corrected Agreement (agreement)

# Chance-Corrected Test re-test reliability (test re-test reliability)

kappa <- (xi-psi)/(1-psi)

}

*The model 3 (with optimistic Beta priors on the two concordant cell rates)*

# Kappa Coefficient (agreement or test re-test reliability)

model{

# Underlying Rates

# Rate “non-respondents” by fMRI indicator (agreement)

# Rate “non-respondents” by first test by OPD (test re-test reliability)

alpha ~ dbeta(2,7)

# Rate ODP decides "non-respondent" when fMRI indicator decides "non-respondent" (agreement)

# Rate re-test decides "non-respondent" when test decides "non-respondent" (test re-test reliability)

beta ~ dbeta(5,1)

# Rate ODP decides "respondent" when fMRI indicator decides "respondent" (agreement)

# Rate re-test decides " respondent" when test decides " respondent" (test re-test reliability)

gamma ~ dbeta(5,1)

# Probabilities For Each Count

pi[1] <- alpha*beta

pi[2] <- alpha*(1-beta)

pi[3] <- (1-alpha)*(1-gamma)

pi[4] <- (1-alpha)*gamma

# Count Data

y[1:4] ~ dmulti(pi[],n)

# Derived Measures

# Rate of ODP agrees with the fMRI indicator (agreement)

# Rate of re-test agrees with the test (test re-test reliability)

xi <- alpha*beta+(1-alpha)*gamma

# Rate of Chance Agreement (agreement)

# Rate of Chance Test re-test reliability (test re-test reliability)

psi <- (pi[1]+pi[2])*(pi[1]+pi[3])+(pi[2]+pi[4])*(pi[3]+pi[4])

# Chance-Corrected Agreement (agreement)

# Chance-Corrected Test re-test reliability (test re-test reliability)

kappa <- (xi-psi)/(1-psi)

}

**References**

1. Lee MD, Wagenmakers EJ,. Bayesian Cognitive Modeling A Practical Course. Cambridge University Press. University Printing House, Cambridge CB2 8BS, United Kingdom (2013).
2. [De Kruijk JR](https://www.ncbi.nlm.nih.gov/pubmed/?term=de%20Kruijk%20JR%5BAuthor%5D&cauthor=true&cauthor_uid=12519649), [Leffers P](https://www.ncbi.nlm.nih.gov/pubmed/?term=Leffers%20P%5BAuthor%5D&cauthor=true&cauthor_uid=12519649), [Menheere PP](https://www.ncbi.nlm.nih.gov/pubmed/?term=Menheere%20PP%5BAuthor%5D&cauthor=true&cauthor_uid=12519649), [Meerhoff S](https://www.ncbi.nlm.nih.gov/pubmed/?term=Meerhoff%20S%5BAuthor%5D&cauthor=true&cauthor_uid=12519649), [Rutten J](https://www.ncbi.nlm.nih.gov/pubmed/?term=Rutten%20J%5BAuthor%5D&cauthor=true&cauthor_uid=12519649), [Twijnstra A](https://www.ncbi.nlm.nih.gov/pubmed/?term=Twijnstra%20A%5BAuthor%5D&cauthor=true&cauthor_uid=12519649). Olfactory function after mild traumatic brain injury. [Brain Inj.](https://www.ncbi.nlm.nih.gov/pubmed/12519649) 2003 Jan;17(1):73-8.
3. Smith BJ. BOA: an R package for MCMC output conver-gence assessment and posterior inference. J Stat Softw2007;21:1—37.
4. Plummer, M. JAGS: A program for analysis of Bayesian graphical models using Gibbs sampling. In Proceedings of the 3rd International Workshop on Distributed Statistical Computing, Vienna, Austria, 20–22 March 2003.
5. Core Team.. R core team. R: A language and environment for statistical computing. Retrieved from <http://www.R-project.org/> (2013)
6. Revelle W. psych: Procedures for Personality and Psychological Research, Northwestern University, Evanston, Illinois, USA, https://CRAN.R-project.org/package=psych Version = 1.7.3. (2017)
7. Su YS, Yajima M. R2jags: using R to Run ‘‘JAGS’’. R PackageVersion 0.5-7. [Accessed on 30 July 2018, available online: http://CRAN.R-project.org/package=R2jags].
8. Plummer M, Best N, Cowles K, Vines K. CODA: Convergence Diagnosis and Output Analysis for MCMC. R News 2006;6:7—11.
9. [Landis JR](https://www.ncbi.nlm.nih.gov/pubmed/?term=Landis%20JR%5BAuthor%5D&cauthor=true&cauthor_uid=843571), [Koch GG](https://www.ncbi.nlm.nih.gov/pubmed/?term=Koch%20GG%5BAuthor%5D&cauthor=true&cauthor_uid=843571). The measurement of observer agreement for categorical data. [Biometrics 1977](https://www.ncbi.nlm.nih.gov/pubmed/843571) Mar;33(1):159-74.
